# Association of gut microbiota dietary index with metabolic dysfunction-associated steatotic liver disease: the mediating roles of inflammation and body mass index

**DOI:** 10.3389/fnut.2025.1573636

**Published:** 2025-05-16

**Authors:** Yu Pu, Zongbiao Tan, Yanrui Wu, Suqi Zeng, Haodong He, Jixiang Zhang, Weiguo Dong

**Affiliations:** ^1^Department of General Practice, Renmin Hospital of Wuhan University, Wuhan, China; ^2^Department of Gastroenterology, Renmin Hospital of Wuhan University, Wuhan, China

**Keywords:** DI-GM, MASLD, hs-CRP, BMI, NHANES

## Abstract

**Background:**

The gut microbiota plays a significant role in the progression of Metabolic Dysfunction-Associated Steatotic Liver Disease (MASLD). The recently introduced Dietary Index of Gut Microbiota (DI-GM), which reflects the diversity of the gut microbiota, has yet to be investigated in relation to MASLD.

**Methods:**

This analysis used raw data from the National Health and Nutrition Examination Survey (2001–2018). MASLD was identified using the US-Fatty Liver Index (US-FLI), and dietary recall data were applied to calculate the Dietary Index of Gut Microbiota (DI-GM). Weighted multivariate logistic regression models assessed the relationship between DI-GM and MASLD. Additionally, mediation analysis was performed to evaluate the influence of high-sensitivity C-reactive protein (hs-CRP) and body mass index (BMI) on the relationship between DI-GM and MAFLD. Propensity score matching (PSM) was employed to minimize confounding and reduce bias inherent to observational studies.

**Results:**

A total of 3,473 participants were included in the analysis, among whom 1,247 were diagnosed with MASLD, with a weighted prevalence of 35.90%. After adjusting for demographic, lifestyle, and metabolic syndrome-related variables, a higher score of DI-GM was strongly linked to a lower risk of MASLD (OR = 0.90, 95% CI: 0.85–0.95, *p* < 0.001). Mediation analysis estimated that BMI accounted for 59.47% of the association (*p* < 0.001), while hs-CRP explained 15.68%. Even after PSM, a higher score of DI-GM remained significantly associated with a lower incidence of MASLD.

**Conclusion:**

The newly proposed DI-GM demonstrated a notable negative correlation with the prevalence of MASLD. Mediation analyses revealed that this relationship was largely influenced by BMI and hs-CRP, highlighting their critical mediating roles.

## 1 Introduction

Metabolic Dysfunction-Associated Steatotic Liver Disease (MASLD) is a chronic liver disorder characterized by excessive hepatic fat accumulation and systemic metabolic dysfunction, independent of significant alcohol intake. It manifests as hepatic steatosis, inflammation, and fibrosis, contributing to liver damage and metabolic disturbances (1). With a rising global prevalence, the prevalence of MASLD in adults is anticipated to exceed 55% by 2040, imposing significant socioeconomic burdens due to its strong links to metabolic and cardiovascular complications (2). Despite growing recognition of its hazards, effective therapeutic options for MASLD are still lacking in clinical practice. Current management relies primarily on lifestyle modifications (3). Therefore, further exploration of its mechanisms is urgent to develop effective treatment and prevention strategies.

Recent studies underscore the pivotal role of diet in influencing gut microbiota composition, which affects metabolic pathways linked to the onset and progression of MASLD (4). Dietary interventions, particularly those emphasizing a diverse intake of fiber-rich foods, polyunsaturated fats, and fermented products, have been shown to positively influence gut microbiota composition, which may provide potential therapeutic avenues for MASLD management (5). Recently, Kase et al. developed a novel dietary index, Diet-Induced Gut Microbiota (DI-GM), which characterizes the relationship between diet and gut microbiota diversity. This index, derived from evidence across 106 longitudinal studies, categorizes foods as beneficial or unfavorable based on their effects on gut microbial diversity, short-chain fatty acid (SCFA) production, and specific bacterial populations (6, 7). Unlike general dietary indices such as the Mediterranean Diet Score or the Healthy Eating Index, DI-GM uniquely targets gut microbiota diversity by integrating detailed evidence on food-bacteria interactions. As a specialized tool, DI-GM provides valuable insights into diet-driven interventions and offers a structured framework for dietary recommendations aimed at improving gut and liver health. Nevertheless, its application in the context of MASLD remains unexplored.

In addition, accumulating evidence implicates chronic inflammation and elevated body mass index (BMI) as pivotal drivers of progression for MASLD. high-sensitivity C-reactive protein (hs-CRP) not only plays a vital role in the pathogenesis of NAFLD, but also serves as an important predictor of NAFLD risk (8, 9). And the association between the severity of MASLD and obesity is also relatively clear (10). Due to the gut microbiota plays an important role in regulating hs-CRP levels and obesity (11, 12), suggesting a potential link between dietary patterns, microbial homeostasis, and the inflammatory/obesity pathway (8, 12, 13). Based on this, we hypothesize that dietary patterns promoting a healthy gut microbiome could mitigate MASLD risk by reducing inflammatory markers and alleviating obesity.

Thus, this study aimed to examine the connection between the DI-GM index and MASLD using data from the National Health and Nutrition Examination Survey (NHANES). Furthermore, we further explored the possible mediating roles of inflammation level (reflected by hs-CRP) and BMI in this relationship.

## 2 Methods

### 2.1 Data sources

NHANES is an ongoing cross-sectional survey aimed at collecting health-related information from a nationally representative sample of the U.S. population that is not institutionalized. The study protocol was approved by the Institutional Review Board of the National Center for Health Statistics, and all participants gave informed consent. Through a multistage probability sampling method, NHANES guarantees the acquisition of comprehensive and trustworthy data, reflecting the health conditions of the U.S. population.

### 2.2 Study design and population

**Figure 1:**
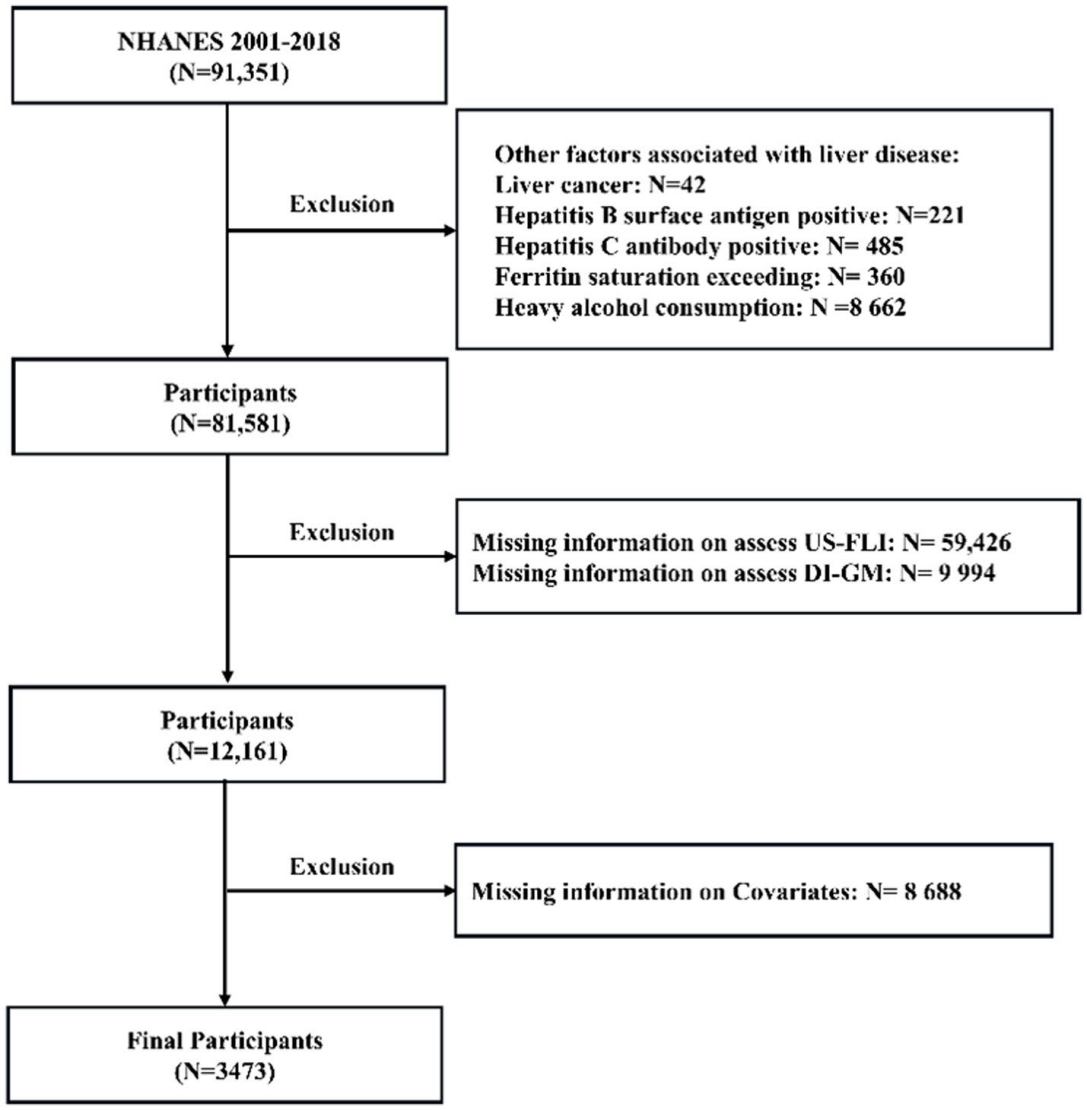
Flowchart for inclusion of study participants from NHANES.

Data were obtained from nine NHANES cycles spanning 2001 to 2018. The exclusion criteria for this study included: ① participants with liver conditions due to other causes; ② *n* = 706: hepatitis viral infection, identified through the detection of hepatitis B surface antigen or confirmation of hepatitis C antibody; ③ *n* = 360: iron overload disorders, characterized by ferritin saturation levels exceeding 50%; and ④ 42: self-reported liver cancer; (2) *n* = 59,426: participants missing data necessary to evaluate MASLD; (3) *n* = 9,994: individuals missing data necessary to evaluate dietary index for gut microbiota, and (4) *n* = 8,688: individuals with incomplete information on other relevant variables. The detailed selection and exclusion process is outlined in Figure 1, providing a clear overview of participant eligibility. An overall total of 3,473 participants who met the inclusion criteria were included in the analysis, consisting of 2,226 non-MASLD patients (64.1%) and 1,247 MASLD patients (35.9%).

### 2.3 Diagnosis of MASLD

Besides ruling out other identifiable causes of liver damage, the diagnosis of MASLD primarily relies on imaging methods such as abdominal ultrasound and magnetic resonance imaging, and other methods for assessing liver fat content. When necessary, a liver biopsy may be performed for further confirmation. However, this method is not widely utilized due to its high operator dependence, cost-related constraints, and its limitation in detecting steatosis only when more than 20–30% of hepatocytes exhibit fat deposition. To address these limitations, CE Ruhl developed a scoring method specifically designed to assess fatty liver in the U.S. population (14). The U.S. Fatty Liver Index (US-FLI) has demonstrated outstanding sensitivity and specificity, and it has been validated in previous research (15). Therefore, this study uses a US-FLI threshold of ≥ 30 as the diagnostic criterion for MASLD.

### 2.4 Assessment of DI-GM

The DI-GM is a composite scoring system designed to evaluate the influence of dietary habits on gut microbiota health, based on evidence linking specific dietary components to gut microbiota diversity and composition. The system comprises 14 dietary components: 10 categorized as beneficial, including fermented dairy, chickpeas, soybeans, whole grains, fiber, cranberries, avocados, broccoli, coffee and green tea, and 4 as unfavorable, including refined grains, red meat, processed meat and high-fat diets (≥40% energy from fat) (6). Dietary recall data from NHANES were used to calculate the DI-GM score. Each component was assigned a binary score: for beneficial components, participants were assigned a score of 1 if their intake exceeded the sex-specific median; otherwise, they received a score of 0. For unfavorable components, a score of 1 was allocated when intake fell below the sex-specific median, except in the case of high-fat diets, where a fixed threshold of 40% of total energy from fat was applied (a score of 1 if below 40%; otherwise, 0). The total DI-GM score, ranging from 0 to 14, was obtained by summing the individual component scores, with higher values indicating dietary habits that are more conducive to gut microbiota health. The total score can be further categorized into subranges: 0–3, 4, 5, and ≥6.

### 2.5 Definition of inflammation level and BMI

Serum hs-CRP levels, quantified by standardized immunonephelometry (Siemens BN-II), served as the inflammation biomarker in this cohort study (16). BMI was determined by dividing body weight (kg) by the square of standing height (m).

### 2.6 Covariates

We obtained all of the variables from the questionnaire, physical examination, and laboratory tests, as detailed in the Supplementary materials. These variables included age, family income-to-poverty ratio (PIR), alanine aminotransferase (ALT), aspartate aminotransferase (AST), Gender, race, education, diabetes mellitus, hypertension, and smoking status (7, 17). Age, PIR, ALT, and AST were treated as continuous variables. Race was classified into four categories: Mexican American, Non-Hispanic Black, Non-Hispanic White, and Others. Education levels were grouped into three categories: less than high school, high school, and some college or an associate degree (AA degree). Smoking status was determined using two questions: “Have you smoked at least 100 cigarettes in your lifetime?” and “Do you smoke now?” Responses categorized participants as never smokers, former smokers, or current smokers.

### 2.7 Statistical analysis

Following the NHANES Analytic Guidelines, this study accounted for the intricate sampling framework and integrated examination sample weights from the mobile examination centers. Descriptive analyses of participants’ baseline characteristics were conducted to compare those with and without MASLD. For continuous variables, the Kolmogorov–Smirnov test is employed to evaluate distributional assumptions. If normality is satisfied, student’s t-test are applied for group comparisons, with results presented as mean ± standard error (SE). For non-normally distributed data (*p* ≤ 0.05), the nonparametric Wilcoxon rank-sum test is utilized, with reported as median with interquartile range (IQR: Q₁–Q₃). Categorical variables were expressed as percentage frequencies (%) and examined using the Chi-square test.

Multivariate logistic regression analysis was performed to examine the association between DI-GM and MASLD across different models. To further explore the relationship between DI-GM and MASLD in specific populations, subgroup analyses were conducted. Interaction analyses were conducted to evaluate potential heterogeneity in associations across different subgroups.

To explore the mediating effects of hs-CRP and BMI in the association between DI-GM and MASLD, we conducted a mediation analysis (18). The models incorporated three key relationships: exposure to the mediator, mediator to outcome, and exposure to outcome (total effect) (19). The total effect was calculated as the sum of the direct effect and the mediated (indirect) effect. The proportion of mediation was determined using the formula: (mediated effect/total effect) × 100%. Additionally, propensity score matching (PSM) is employed for sensitivity analysis to confirm the robustness of the study’s findings.

All analyses were performed using R (version 4.2.3). The “survey” package (version 4.4.2) was employed for survey sample analysis, the “mediation” package (version 4.5.0) for mediation analysis, and the “MatchIt” package (version 4.5.5) for PSM. Statistical significance was defined by two-sided *p*-values < 0.05.

## 3 Results

### 3.1 Characteristics of the participants

Table 1 provides an overview of the demographic and health characteristics of the study participants, consisting of 3,473 individuals with a median age of 53 years. Of these, 1,247 were diagnosed with MASLD, with a prevalence of 35.90%. Compared to those without MASLD, individuals with MASLD were more likely older, males, Mexican descent, and had lower levels of income and education. Meanwhile, they exhibited lower DI-GM scores, higher level of HOMA-IR, BMI, ALT, AST, and hs-CRP. Moreover, participants with MASLD were significantly more likely to have hypertension and diabetes than those without MASLD.

**Table 1:** Characteristics of the study participants.

Variables	Total (*n* = 3,473)	Non-MASLD (*n* = 2,226)	MASLD (*n* = 1,247)	*p*
Age, M (Q₁, Q₃)	53.00 (39.00, 67.00)	50.00 (35.00, 65.00)	58.00 (45.00, 70.00)	<0.001
PIR, M (Q₁, Q₃)	2.20 (1.17, 4.29)	2.40 (1.25, 4.44)	1.99 (1.09, 3.92)	<0.001
BMI, M (Q₁, Q₃)	27.99 (24.36, 32.30)	25.87 (23.01, 29.20)	32.18 (28.80, 36.39)	<0.001
DI GM, M (Q₁, Q₃)	5.00 (4.00, 6.00)	5.00 (4.00, 6.00)	4.00 (3.00, 6.00)	<0.001
Alt, M (Q₁, Q₃)	21.00 (16.00, 28.00)	19.00 (15.00, 24.00)	25.00 (20.00, 34.00)	<0.001
Ast, M (Q₁, Q₃)	23.00 (20.00, 27.00)	23.00 (19.00, 26.00)	25.00 (21.00, 30.00)	<0.001
hs-CRP, M (Q₁, Q₃)	0.19 (0.08, 0.45)	0.14 (0.06, 0.33)	0.31 (0.15, 0.67)	<0.001
HOMA IR, M (Q₁, Q₃)	2.64 (1.59, 4.62)	1.83 (1.28, 2.65)	5.45 (3.95, 7.79)	<0.001
Gender, n (%)				<0.001
Female	1867 (53.76)	1,291 (58.00)	576 (46.19)	
Male	1,606 (46.24)	935 (42.00)	671 (53.81)	
Race, n (%)				<0.001
Black	644 (18.54)	508 (22.82)	136 (10.91)	
Mexican	553 (15.92)	254 (11.41)	299 (23.98)	
Other	506 (14.57)	336 (15.09)	170 (13.63)	
White	1770 (50.96)	1,128 (50.67)	642 (51.48)	
Education, n (%)				<0.001
High school	1,273 (36.65)	789 (35.44)	484 (38.81)	
Less than high school	432 (12.44)	204 (9.16)	228 (18.28)	
Some college or AA degree	1768 (50.91)	1,233 (55.39)	535 (42.90)	
Diabetes Mellitus, n (%)				<0.001
No	2,685 (77.31)	1926 (86.52)	759 (60.87)	
Yes	788 (22.69)	300 (13.48)	488 (39.13)	
Hypertension, n (%)				<0.001
No	1912 (55.05)	1,415 (63.57)	497 (39.86)	
Yes	1,561 (44.95)	811 (36.43)	750 (60.14)	
Smoke, n (%)				<0.001
Former	926 (26.66)	505 (22.69)	421 (33.76)	
Never	1998 (57.53)	1,338 (60.11)	660 (52.93)	
Now	549 (15.81)	383 (17.21)	166 (13.31)	

The DI-GM ranges from 0 to 14 and grouped according to 0–3, 4, 5, and ≥6. DI-GM, dietary index for gut microbiota; NHANES, National Health and Nutrition Examination Survey; PIR, Poverty Income Ratio; BMI, Body Mass Index; hs-CRP, high-sensitivity C-reactive protein.

### 3.2 Association between DI-GM and MASLD

As shown in Table 2, a negative correlation between DI-GM (as a continuous variable or categorical variable) and the occurrence of MASLD was found in all models (including the crude and adjusted models). Specifically, each one-point increase in DI-GM corresponded to a 10% reduction in the risk of MASLD (OR = 0.90, [95% CI: 0.85, 0.95]) in crude model. The above associations remained significant in the model 1 (MASLD: OR = 0.89, [95% CI: 0.84, 0.94]) and model 2 (MASLD: OR = 0.91, [95% CI: 0.85, 0.98]). When DI-GM was converted from a continuous to a categorical variable, in the model 1, participants with DI-GM = 5 were significantly negatively correlated with the risk of MASLD (OR = 0.77, [95% CI: 0.61, 0.98]), and those with a DI-GM ≥ 6 showed an even stronger negative correlation with MASLD risk in both model 1 [OR = 0.57, (95% CI: 0.45, 0.72)] and model 2 [OR = 0.62, (95% CI: 0.45, 0.86)].

**Table 2:** Association between DI-GM and MASLD of the NHANES 2001–2018 participants.

Characteristics	Crude model		Model 1		Model 2	
	95%CI	*p*	95%CI	*p*	95%CI	*p*
DI-GM	0.90 (0.85, 0.95)	<0.001	0.89 (0.84,0.94)	<0.001	0.91 (0.85, 0.98)	0.02
Character				
0–3	Ref		Ref		Ref	
4	0.89 (0.69, 1.14)	0.336	0.89 (0.67,1.17)	0.384	0.95 (0.72, 1.26)	0.728
5	0.75 (0.61, 0.93)	0.012	0.77 (0.61,0.98)	0.038	0.88 (0.67, 1.17)	0.36
≥6	0.61 (0.48, 0.77)	<0.001	0.57 (0.45,0.72)	<0.0001	0.62 (0.45, 0.86)	0.01
Trend test		<0.0001		<0.0001		0.005

Cruel model: DI_GM4. Model 1: Adjusted for age, gender, race, education, PIR. Model 2: adjusted, age, gender, race, education, Alt, Ast, Hypertension, hs-CRP. Ref, reference. *p* value in bold indicates statistical significance. MASLD, Metabolic Dysfunction-Associated Steatotic Liver Disease; DI-GM, dietary index for gut microbiota; PIR, Poverty Income Ratio; hs-CRP, high-sensitivity C-reactive protein, Body Mass Index.

### 3.3 Association between DI-GM and MASLD among different subgroups

The subgroup analyses were performed to further evaluate the reliability and robustness of the association between DI-GM and MASLD across various demographic and health-related subgroups. The summarized results for subgroup analyses were presented in Table 3 align with the overall findings in the full population. In particular, a more pronounced decline in MASLD was observed among women, individuals of Mexican or Caucasian ancestry, those with higher educational and income levels, and those without diabetes. Additionally, our findings suggest that the higher level of DIGM was associated with a lower risk of MASLD, regardless of hypertension status. There is no significant interaction effects were detected.

**Table 3:** Association between DI-GM and MASLD by different group stratification.

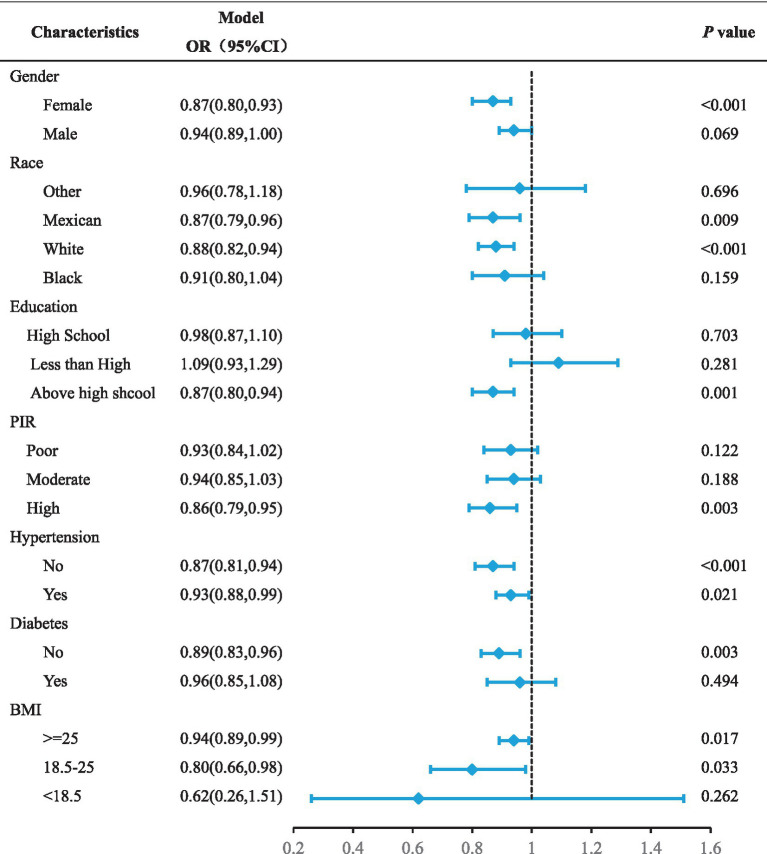

CI, Confidence interval; DI-GM, dietary index for gut microbiota; NHANES, National Health and Nutrition Examination Survey; OR, Odd Ratio; PIR, poverty income ratio; BMI, body mass index.

### 3.4 Mediation and additional analysis

The relationship between the index DI-GM and MASLD using hs-CRP and BMI as mediating variables as shown in the Table 4. Both the direct and indirect effects of DI-GM on the prognosis of MASLD, mediated by hs-CRP, were statistically significant (*p* < 0.001), indicating that part of the effect of DI-GM on MASLD may be mediated through hs-CRP levels. Specifically, hs-CRP accounts for approximately 15.68% of the total association between the DI-GM and MASLD (*p* < 0.001). In contrast, BMI was found to play a more substantial mediating role. While the direct effect of DI-GM on MASLD through BMI was not statistically significant (*p* = 0.060), the indirect effect was highly significant (*p* < 0.001), with BMI mediating approximately 59.47% of the total association between DI-GM and MASLD (*p* < 0.001). This highlights BMI as a key factor in explaining the relationship between DI-GM and MASLD.

**Table 4:** Mediation effect of hs-CRP and BMI on the association between DI-GM and MASLD.

Mediator		Direct effect	Indirect effect (Mediated)	Total effect	Proportion mediated (%)
hs-CRP	Estimate	−0.019	−0.003	−0.022	15.68%
	95% *CI*	(−0.029, −0.010)	(−0.006, 0)	(−0.033, −0.010)	
	*P* value	<0.001	<0.001	<0.001	<0.001
BMI	Estimate	−0.009	−0.013	−0.021	59.47%
	95% *CI*	(−0.018, 0)	(−0.018, −0.010)	(−0.032, −0.010)
	*P* value	0.060	<0.001		<0.001	<0.001

Models were adjusted for age, sex, race/ethnicity, education level, PIR, Alt, Ast, and hypertension. Abbreviations: MASLD: Metabolic Dysfunction-Associated Steatotic Liver Disease; CI, Confidence Interval; DI-GM, dietary index for gut microbiota, PIR, poverty income ratio; BMI, body mass index; hs-CRP, high-sensitivity C-reactive protein.

### 3.5 Propensity score matching analysis

To minimize the impact of confounding factors and eliminate the bias of the observational study, a 1:1 PSM was performed. There was no statistically significant difference was found between Non-MASLD and MASLD participants in terms of the covariates. However, the diabetes patients were found to be higher than in non-MASLD PSM controls (*p* < 0.05) in the Supplementary Table 1. In the logistic regression analysis performed after PSM in the Supplementary Table 2, it was observed that DI-GM was significantly linked with the outcome. Specifically, in the crude model, DI-GM showed a protective effect with an OR of 0.93 (95% CI: 0.87–0.99, *p* = 0.02), and this association remained statistically significant after adjustment for confounders in Model 1, with an OR of 0.94 (95% CI: 0.88–1.00, *p* = 0.04).When stratified by the characteristic variable, individuals with a score of ≥6 showed a significantly reduced risk compared to the reference group (0–3), with an OR of 0.65 (95% CI: 0.49–0.87, *p* = 0.005) in the crude model and 0.67 (95% CI: 0.50–0.90, *p* = 0.011) in Model 1 after adjustment. However, no statistically significant associations were found for groups with scores of 4 or 5 in either model. Additionally, a trend test revealed a significant negative association between higher characteristic scores and the outcome in both the crude model (*p* = 0.003) and Model 1 (*p* = 0.01), indicating a dose–response relationship. These findings suggest that higher scores or levels of DI-GM may be protective, even after adjusting for potential confounding variables.

## 4 Discussion

Our study demonstrated that individuals with MASLD were more likely to be older, male, Mexican descent and have lower income and education levels. They also exhibited higher levels of HOMA-IR, BMI, ALT, AST, and hs-CRP. Furthermore, participants with MASLD had a significantly higher prevalence of hypertension and diabetes compared to those without MASLD. These findings align with previous research suggesting that aging, male, unhealthy lifestyle, metabolic syndrome, and increased exposure to health risks contribute to the exacerbation of MASLD (20–23). Crucially, our study is the first to show that DI-GM scores and the prevalence of MASLD were negatively correlated. Mediation analysis further revealed BMI and hs-CRP as key mechanistic mediators linking DI-GM to MASLD pathogenesis.

Diet is a key determinant of gut microbiota composition, shaping microbial diversity and abundance through both direct and indirect mechanisms. Previous study has shown that high-fat diets can induce gut microbiota dysbiosis, leading to the production of intestinal metabolites such as lipopolysaccharides, ultimately contributing to chronic low-grade liver inflammation (24, 25). The Mediterranean diet, high-fiber diet, ketogenic diet, intermittent fasting and consumption of fermented foods reduce oxidative stress and hepatic inflammation, while enhancing beneficial gut microbial populations and modulating gut microbiota, thereby offering therapeutic potential for MAFLD (26–31).Although these studies have investigated the effects of specific dietary components or patterns on gut microbiota in MASLD populations, a comprehensive dietary measurement score closely associated with gut microbiota diversity and abundance remains absent. A novel dietary index, DI-GM, addresses this gap (6). Our study revealed a significant inverse association between higher DI-GM scores (particularly scores ≥6, indicating greater intake of beneficial dietary components) and risk of MASLD. Among the beneficial components, fermented dairy products increase Lactobacillus populations, alleviating gut dysbiosis (32, 33).A cross-sectional study found that increased yogurt intake was dose-dependently associated with a reduced incidence of newly diagnosed NAFLD, which is consistent with our findings (34).Chickpeas and soybeans stimulate the growth of Bifidobacteria (35, 36), and dietary pulse intake improves gut flora induced by a high-fat diet and is associated with a reduction in the incidence of metabolic dysfunction-related diseases, including MAFLD (37, 38).Whole grains and fiber enhance the beneficial gut flora (39), which is consistent with previously proposed guidelines for lifestyle interventions in NAFLD (40). In addition, coffee polyphenols and green tea catechins act as prebiotics to enhance microbial proliferation and balance gut microbiota (41, 42). And meta-analyses have shown that green tea and coffee drinkers have a lower risk of developing NAFLD (43, 44).

Probiotics, beneficial microorganisms that counteract harmful bacteria, represent a promising, cost-effective, and accessible therapeutic option for the treatment of MASLD. Several clinical studies have shown that different probiotic strains can prevent and improve NAFLD by influencing various biomarkers (45–47). Some RCTs have shown that the use of probiotics can lead to weight loss and a reduction in BMI in patients with NAFLD (48, 49). Additionally, probiotics have been associated with potential benefits for metabolic parameters associated with NAFLD, including decreased inflammatory markers (50). In some studies, taking symbiotic bacteria was found to show reductions in most inflammatory mediators, including CRP, TNF-αand IL-6 (50–52). Overall, probiotic supplementation may offer a safe adjunctive therapy for MAFLD by modulating gut microbiota composition, improving epithelial barrier function, and enhancing immune system regulation (53).

In our study, BMI and hs-CRP were identified as the mediator of the relationship between DI-GM and MASLD. This is consistent with the previous description that intake of foods that are beneficial in regulating gut flora or increase prebiotics, can reduce the risk of MASLD by lowering hs-CRP and BMI. The higher DI-GM indicates a healthy gut microbiome that helps maintain good metabolic function and reduces inflammation levels in the body, potentially reducing the risk of MASLD. These evidence highlights the interplay and communication between the liver and gut, particularly the gut microbiota, as a key factor in the metabolic dysregulation and inflammation characteristic of MASLD (1, 54).

This study has several limitations that should be recognized. First, the cross-sectional design of our analysis limits the ability to establish causality between DI-GM and MASLD. Although our findings provide valuable insights, prospective studies and randomized controlled trials are required to confirm these associations. Additionally, like many observational studies, the potential for residual confounding cannot be fully excluded. Measurement errors in unmeasured variables or the influence of unknown confounders may have affected our results. Lastly, the reliance on self-reported 24-h dietary recall data and certain self-reported covariates, such as lifestyle factors, introduces the possibility of recall bias and may reduce data accuracy. Future studies could enhance causal inference by incorporating direct gut microbiota profiling, long-term follow-up data, and more objective measures of dietary intake and covariates.

## 5 Conclusion

This study highlights the negative association between the newly developed DI-GM, a dietary quality index linked to gut microbiota diversity, and the prevalence of MASLD. Mediation analyses revealed that hs-CRP and BMI play significant roles in this relationship, underscoring the complex interplay between diet, inflammation, and metabolism. Given the strong connections among diet, gut microbiota, and MASLD, future research should focus on validating these findings in diverse populations and developing targeted dietary interventions. Such efforts could provide practical strategies to reduce the burden of MASLD globally.

## Data Availability

The original contributions presented in the study are included in the article/Supplementary material, further inquiries can be directed to the corresponding author.
