# Supplementary material for: Association of gut microbiota dietary index with metabolic dysfunction-associated steatotic liver disease: the mediating roles of inflammation and body mass index
Source: Front Nutr. 2025 May 16;12:1573636. doi: 10.3389/fnut.2025.1573636 (PMC12123378; doi:10.3389/fnut.2025.1573636)
Supplement: Supplementary file 1 [file Table_1.docx]

Supplementary Table 1. Basic characteristics of participants with DI-GM with/without MASLD after PSM Analysis

| Variable | Total  (n = 2168) | Non-MASLD  (n = 1084) | MASLD  (n = 1084) | *P* |
| --- | --- | --- | --- | --- |
|  |  |  |  |  |
| Age, M (Q₁, Q₃) | 57.00 (42.00, 70.00) | 58.00 (41.00, 71.00) | 57.00 (44.00, 69.00) | 0.799 |
| PIR, M (Q₁, Q₃) | 2.06 (1.11, 3.98) | 2.12 (1.13, 3.92) | 2.02 (1.09, 4.08) | 0.348 |
| Gender, n (%) |  |  |  | 0.323 |
| Female | 1057 (48.75) | 540 (49.82) | 517 (47.69) |  |
| Male | 1111 (51.25) | 544 (50.18) | 567 (52.31) |  |
| Race, n (%) |  |  |  | 0.967 |
| Black | 277 (12.78) | 142 (13.10) | 135 (12.45) |  |
| Mexican | 462 (21.31) | 228 (21.03) | 234 (21.59) |  |
| Other | 305 (14.07) | 153 (14.11) | 152 (14.02) |  |
| White | 1124 (51.85) | 561 (51.75) | 563 (51.94) |  |
| Education, n (%) |  |  |  | 0.565 |
| High School | 841 (38.79) | 419 (38.65) | 422 (38.93) |  |
| Less than High School | 334 (15.41) | 159 (14.67) | 175 (16.14) |  |
| Some College or AA degree | 993 (45.8) | 506 (46.68) | 487 (44.93) |  |
| Diabetes, n (%) |  |  |  | 0.013 |
| No | 1548 (71.4) | 800 (73.80) | 748 (69.00) |  |
| Yes | 620 (28.6) | 284 (26.20) | 336 (31.00) |  |
| Hypertension, n (%) |  |  |  | 0.605 |
| No | 982 (45.3) | 497 (45.85) | 485 (44.74) |  |
| Yes | 1186 (54.7) | 587 (54.15) | 599 (55.26) |  |
| Smoke, n (%) |  |  |  | 0.519 |
| Former | 659 (30.4) | 319 (29.43) | 340 (31.37) |  |
| Never | 1210 (55.81) | 618 (57.01) | 592 (54.61) |  |
| Now | 299 (13.79) | 147 (13.56) | 152 (14.02) |  |

**Abbreviations:** MASLD: Metabolic Dysfunction-Associated Steatotic Liver Disease; DI-GM, dietary index for gut microbiota; NHANES, National Health and Nutrition Examination Survey; PIR, Poverty Income Ratio; PSM, Propensity Score Matching;

Supplementary Table 2. Association between DI-GM and MASLD of the NHANES 2001-2018 participants after PSM.

| Characteristics | Crude model | |  | Model1 | | |
| --- | --- | --- | --- | --- | --- | --- |
|  | 95%CI | *P* |  | | 95%CI | *P* |
| DI-GM | 0.93(0.87,0.99) | 0.02 |  | | 0.94(0.88,1.00) | 0.04 |
| character |  |  |  | |  |  |
| 0-3 | Ref |  |  | | Ref |  |
| 4 | 0.91(0.62,1.33) | 0.605 |  | | 0.93(0.63,1.38) | 0.72 |
| 5 | 0.91(0.65,1.27) | 0.564 |  | | 0.99(0.71,1.37) | 0.937 |
| >=6 | 0.65(0.49,0.87) | 0.005 |  | | 0.67(0.50,0.90) | 0.011 |
| Trend test |  | 0.003 |  | |  | 0.01 |

Abbreviations: CI, Confidence Interval; DI-GM, dietary index for gut microbiota; NHANES, National Health and Nutrition Examination Survey; OR, Odd Ratio;

^a^. The crude model was not adjusted for any covariates, while model 1 was adjusted for Alt and Ast

^b^. The DI-GM ranges from 0-14 and grouped according to 0-3, 4, 5, and ≥6.
